# Bilateral Subacromial-Subdeltoid Rice Bodies in the Shoulder: A Surgical Case Report

**DOI:** 10.1155/2024/8299485

**Published:** 2024-04-03

**Authors:** Caroline Rush, Olivia Jochl, Natalie Lowenstein, Jillian Mazzocca, Elizabeth Matzkin

**Affiliations:** ^1^Science Division, Tufts University School of Medicine, Boston, Massachusetts, USA; ^2^Research Division, Department of Orthopaedic Surgery, Brigham and Women's Hospital, Boston, Massachusetts, USA; ^3^Department of Public Health, Sidney Kimmel Medical College, Thomas Jefferson University, Philadelphia, PA, USA; ^4^Women's Sports Medicine, Surgery Division, Department of Orthopaedic Surgery, Brigham and Women's Hospital, Boston, Massachusetts, USA

## Abstract

**Introduction:**

Rice bodies (RBs) are pale and glossy appearing small fibrinous nodules that form due to synovial or tenosynovial joint inflammation. RBs are significant as they are common in orthopedic practices causing nonspecific symptoms such as pain, swelling, range of motion limitations, crepitus, and catching sensations. These loose bodies occur often within the bursa as a symptom of chronic bursitis and are commonly associated with rheumatoid, inflammatory, or tuberculous arthritis. Reports on RBs are present; however, few bilateral cases within the shoulder appear in the literature. *Case Presentation*. This case demonstrates an unusual bilateral, subacromial-subdeltoid presentation of rice bodies (RBs) in a 41-year-old Caucasian female patient with a history of rheumatoid arthritis. We describe treatment with right shoulder arthroscopy to remove the loose bodies. One-year postoperative patient-reported outcomes (PROMs) show improvement in symptoms, pain, and overall function.

**Conclusion:**

Formation of RBs occurs as a symptom of an inflammatory response in synovial joints. This provokes multiple small fibrin aggregates to collect within synovial bursae and occasionally tendon sheaths. RBs are rarely seen bilaterally. Arthroscopic removal of RBs is an appropriate treatment method for symptom improvement.

## 1. Introduction

Rice bodies (RBs) are pale and glossy appearing small fibrinous nodules that form due to synovial or tenosynovial joint inflammation. Nodule formation may be the result of synovial inflammation, tissue microinfarction, and subsequent shedding, producing the nodule's core of fibrinous necrosis, chronic granulomatous inflammatory cells, and collagen that is then encased in the fibrin within the synovial fluid [1, 2]. This pathophysiology hypothesis suggests such synovial element aggregation to be preceded by a proinflammatory environment. Alternatively, the nodules may form independent of the synovium simply as fibrin aggregates [3]. Regardless of the pathophysiology of their formation, these nodules do not contain cartilaginous material.

RBs are not uncommon to orthopedic practices causing nonspecific symptoms such as pain, swelling, range of motion limitations, crepitus, and catching sensations. These loose bodies occur often, but not always, within the bursa as a symptom of chronic bursitis and are commonly associated with rheumatoid, inflammatory, or tuberculous arthritis [4]. Reports of RBs include presentations in the knee and shoulder in addition to rare cases presenting in the wrist and elbow [5, 6]. Most cases present unilaterally, though few bilateral cases within the shoulder and wrist have been reported [7–9].

This report describes the unusual presentation of bilateral subacromial-subdeltoid RB formation in a 41-year-old female patient. Rice body clinical management includes treating any suspected underlying causes in addition to removal of the loose bodies and synovectomy procedures [10].

## 2. Case Presentation

Informed consent was given by the patient for the publication of this report and associated figures. A 41-year-old female presented to the clinic with a one-year history of persistent right shoulder pain and crepitus. She presented to rheumatology six months prior with diffuse polyarticular arthritic pain that had not improved with two months of physical therapy. She was then diagnosed with rheumatoid arthritis (RA). Most of her joint pain including that of her neck, left shoulder, and proximal interphalangeal and metacarpophalangeal joints was attenuated with hydroxychloroquine with the exception of her right shoulder.

On baseline physical examination of the right shoulder, diffuse tenderness was noted, along with positive impingement tests. Range of motion was lacking by approximately 10° in abduction and flexion in both the right and left shoulders but was otherwise normal. These physical exam findings provide utility in understanding the symptomatic severity of the disease and increase the threshold to image the joint for further diagnostic certainty.

**Figure 1:**
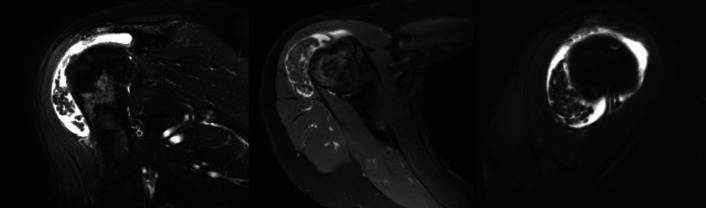
Right shoulder MRI. Magnetic resonance imaging (MRI) of the right shoulder of the patient preoperatively. The figure depicts three MRI images revealing multiple loose bodies within the subacromial space consistent with RB and a small, nontraumatic partial tear in the supraspinatus tendon, along with subacromial and subdeltoid bursitis.

Magnetic resonance imaging (MRI) was obtained by an outside institution. It showed multiple loose bodies within the subacromial space consistent with RBs and a small, nontraumatic partial tear in the supraspinatus tendon, along with subacromial and subdeltoid bursitis (Figure 1).

After exhausting nonoperative treatment measures, including activity modification, anti-inflammatories, and physical therapy, the patient opted for a right shoulder arthroscopy to remove the loose bodies, resect the bursa, and repair the partial rotator cuff tear.

**Figure 2:**
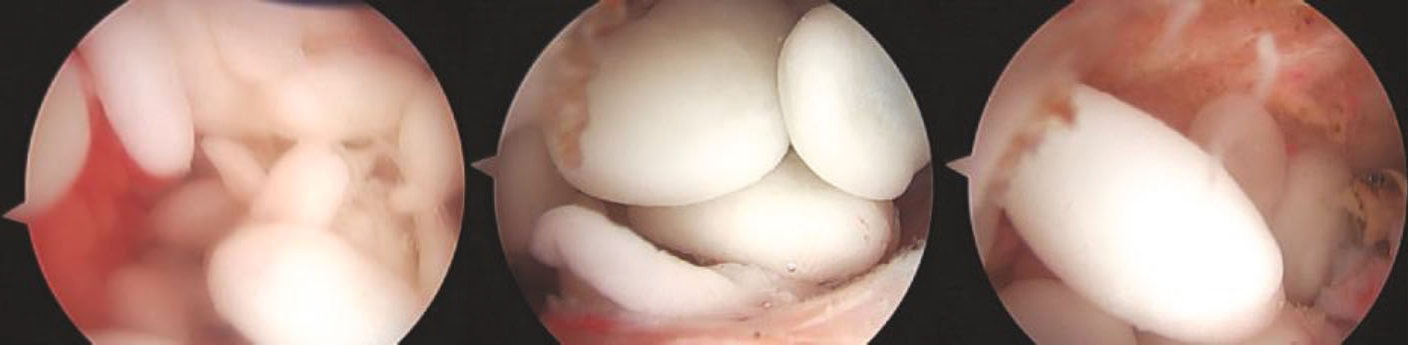
Right shoulder arthroscopic images. Three intraoperative images from the patient of interest of the rice bodies in the subacromial space of the right shoulder.

At the time of arthroscopy, the glenohumeral compartment was free of any loose bodies. Upon insertion of the arthroscope into the subacromial space, multiple loose bodies were visualized. Subacromial bursectomy and loose body removal were performed arthroscopically. Several bodies were sent to pathology for analysis. The presence of RBs was confirmed, as the analysis revealed nodules of fibrinous material with focal hyalinization and no cartilaginous/chondroblastic differentiation. Numerous loose bodies were removed from the subacromial space (Figure 2). Finally, during the procedure, a partial-thickness tear of the supraspinatus tendon from the bursal side was confirmed and repaired using a standard rotator cuff repair technique.

The patient began physical therapy 3 weeks postsurgery and, at 3 months postoperatively, was happy with her rehabilitation progress. She was continuing to improve her range of motion as well as strength at the expected rate. During this time, the patient noted increased soreness and swelling in her contralateral shoulder with no known history of injury. She disclosed increased pain in her left shoulder when undergoing daily tasks, including, but not limited to, getting dressed and doing her hair. While completing her right shoulder rehabilitation, she was also working with physical therapy for her left shoulder but saw no improvement and experienced worsening pain, clicking, and catching. As most cases of rice bodies are reported unilateral, there was no reason to suspect bilateral pathology prior to this persistent pain.

**Figure 3:**
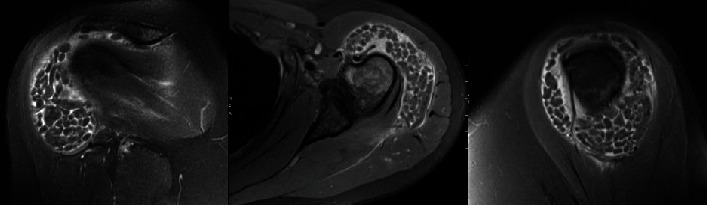
Left shoulder MRI. Magnetic resonance imaging (MRI) of the left shoulder of the patient preoperatively. The figure depicts three MRI images revealing loose bodies within the subacromial and subdeltoid bursa.

**Figure 4:**
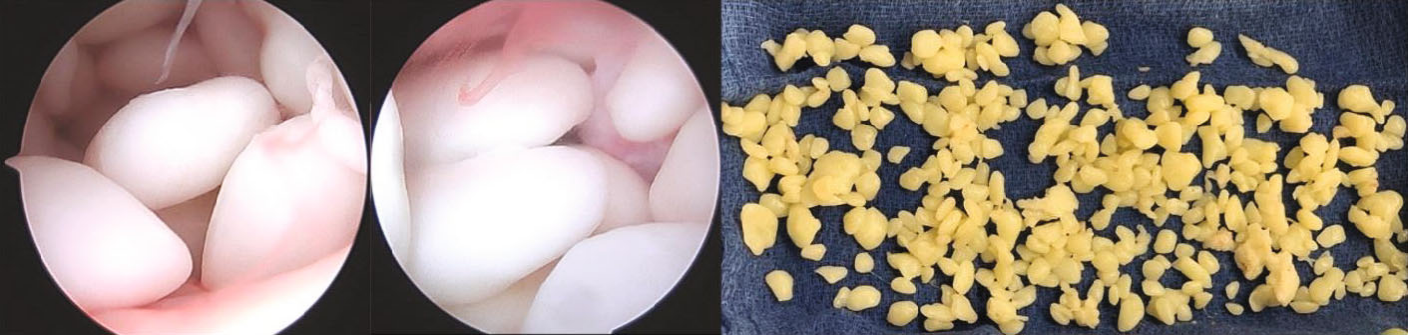
Left shoulder arthroscopic images and loose bodies removed from the left shoulder. The first two images in this figure reveal intraoperative pictures from the patient of interest of the rice bodies in the left shoulder. The last image shows the loose rice bodies succeeding extraction.

An MRI of the left shoulder was performed and revealed similar loose bodies within the subacromial and subdeltoid bursa (Figure 3). The patient opted for shoulder arthroscopy due to progressive pain, swelling, and crepitus. Arthroscopy of the left shoulder was performed and demonstrated no other pathology within the glenohumeral compartment. Upon visualization of the subacromial space, hundreds of loose bodies, with the largest measuring over ten millimeters, were visualized (Figure 4). Prolific bursitis was also identified. The loose bodies were removed, and a sample was sent to pathology for analysis, once again confirming RB pathology.

The patient completed her postoperative rehabilitation protocols for both shoulders, and at her last postoperative visit, she was 1.5 years from her right shoulder arthroscopy and 1 year from her left shoulder arthroscopy. At this time, she reported full range of motion, normal strength, and no pain with her activities or at night. Preoperative and postoperative patient-reported outcome measures (PROMs) are reported in Table 1.

## 3. Discussion

We present an unusual case of bilateral extra-articular RBs of the shoulder in a female patient. Rice bodies seen in the shoulder are commonly found in the subacromial and subdeltoid bursa, but few reported cases are bilateral [6–8]. Literature documenting RBs in the shoulder is summarized in Table 2. Table 2 demonstrates that RB formation in the shoulder occurs more commonly in female patients and often cooccurs with rheumatologic disorders, specifically rheumatoid arthritis. We suspect that the patient's history of RA impacted the development and progression of RBs. Two cases reported administering two corticosteroid injections either alone or prior to excisional treatment. In one case, the injections provided minimal-to-moderate relief while the other case demonstrated complete resolution of symptoms from the injections [11, 12]. Successful corticosteroid use to mitigate symptoms supports the idea that rice bodies are a consequence of an inflammatory process. Additionally, two cases of RBs in the shoulder have occurred following rotator cuff surgeries possibly due to polylactic acid implants [13, 14]. Neither of these cases had other underlying relevant pathology, which further demonstrates RB development to be an inflammatory reaction that does not require underlying intrinsic pathology like a rheumatologic disorder to initiate the disease process.

The differential diagnosis for rice bodies (RBs) includes the similarly appearing loose bodies formed in synovial chondromatosis (SC). Although almost identical in presentation, loose bodies formed in SC differ dramatically from rice bodies in their composition as they are composed of unmineralized or mineralized metaplastic cartilage as opposed to fibrin [4]. The clinical presentation of RB formation is often nonspecific and could be conflated with SC; thus, diagnosis requires further imaging. X-ray fails to show nodules that have not calcified, so MRI is required for definitive findings and is best used by comparing the apparent intensity of a nodule to that of skeletal muscle. T1-weighted images exhibit unmineralized SC nodules with a slightly hyperintense signal, mineralized SC nodules with a hypointense signal, and RBs with an isointense signal, rendering RBs indistinguishable in T1-weighted imaging. Alternatively, T2-weighted imaging will make SC nodules hyperintense if unmineralized or hypointense if mineralized and RBs slightly hyperintense [4]. Therefore, T1-weighted imaging tends to be useful in confirming RB formation as the diagnosis in addition to its distinguishing pathology.

Although RBs are benign, they can significantly impact a patient's quality of life and cause further articular and periarticular damage. Therefore, it is important to begin treatment as early as possible and consider surgical intervention once nonoperative options have been exhausted [11]. Arthroscopic management of RBs can alleviate pain as well as symptoms, and removal of the loose bodies can help prevent progression of concomitant arthritis and rotator cuff tears [7, 16]. Arthroscopy, rather than open arthrotomy, provides the opportunity for good visualization during surgery, low morbidity, early return to activity, and early healing. A bursectomy or synovectomy is often performed due to bursal wall inflammation with dense lymphoplasmacytic infiltration of villous hyperplastic synovium lining the sac [4, 8].

The understanding of this case is limited by the sparse reports of rice bodies in the current literature and the extent of this case's pathology report. Further investigation into the epidemiology and pathophysiology of rice bodies will elucidate their correlation with proinflammatory physiology as well as certainty of recurrence and treatment. This case particularly necessitates inquiry into oligoarticular rice body formation and its precipitating factors.

## 4. Conclusion

Joint inflammation with concomitant rice body formation is a rare but symptomatic and treatable clinical presentation. Appropriate treatment for RB formation in the shoulder includes managing any associated underlying disease in addition to arthroscopic removal of the loose bodies for symptom relief. This remarkably uncommon case of bilateral RBs demonstrates the efficacy of this treatment and impels a further understanding of the mechanism of this disease.

### 4.1. Clinical Message

This report presents a case of bilateral subacromial, subdeltoid rice bodies as diagnosed using pathology in addition to clinical symptoms and evaluation. Loose body diagnostic certainty requires pathologic investigation. More precise diagnosis of loose bodies will contribute to an improved understanding of rice body pathophysiology and approach to prognosis and treatment. Correct and accurate identification of these loose bodies will equip physicians with the knowledge required to treat patients in the most optimal capacity.

## Figures and Tables

**Table 1:** Outcome scores at pre-Op and 1-year post-Op. Preoperative and postoperative patient-reported outcome measures of the patient analyzed in this case report.

PROM	Right shoulder		Left shoulder	
	Pre-Op	1-year post-Op	Pre-Op	1-year post-Op
ASES Shoulder Assessment	75.83	87.53	87.50	94.20
SANE	60	83.0	43.0	83.0
SST	75	83.33	66.67	100.0
VAS	2.5	1.16	0.5	0.16
VR-12				
Physical	48.16	52.06	44.04	46.62
Mental	44.31	44.73	43.46	51.23

Note: ASES: American Shoulder and Elbow Surgeons; SANE: Single Assessment Numeric Evaluation; SST: Simple Shoulder Test; VAS: visual analog pain scale; VR-12: Veterans Rand 12-item Health Survey.

**Table 2:** Literature reporting on rice bodies in the shoulder.

Author, year	Sex	Age	Location	Laterality	Sx dur.	Underlying dx	Tx	Recur
This case, 2023	Female	41	Subacromial, subdeltoid	Bilat	1 yr	RA	RB removal, bursectomy	No
Law, 1998 [8]	Female	31	Subacromial, subdeltoid	Bilat	5 yr	RA	RB removal, bursectomy	No
Steinfeld, 1994 [7]	Female	45	Subacromial, subdeltoid	Bilat	Unknown	ANA+	Bursectomy	No
Steinfeld, 1994 [7]	Male	38	Subacromial	Right	2.5 yr	None	Bursectomy	No
Steinfeld, 1994 [7]	Male	71	Subacromial	Right	2 yr	RF+	Bursectomy	No, RA
Cuomo, 2006 [15]	Male	4	Biceps tenosynovium	Right	3 mo	Juvenile RA	Bursectomy	No
Joshi, 2018 [2]	Female	54	Subacromial	Right	5 yr	RA	RB removal, bursectomy	No
Guo, 2020 [1]	Female	27	Subacromial, subdeltoid	Right	2 yr	RA	RB excision, bursa & “red tissue” ablation	No
Guo, 2020 [1]	Female	45	Subacromial, subdeltoid	Left	2 yr	Hx of breast cancer and radiotherapy, RF+	RB excision, bursa & “red tissue” ablation	No
Vyas, 2022 [12]	Female	29	Subacromial subdeltoid	Right	2 yr	RA	Injected with 80 mg methylprednisolone acetate under ultrasound guidance ×2	No
Best, 2015 [11]	Female	52	Subacromial subdeltoid	Right	2 mo	RA	Injection ×2, removal consult	Unknown
Barad, 2019 [13]	Male	48	Subacromial subdeltoid	Left	0 mo	Polylactic acid staples	Debridement and drainage	No
Sivaloganathan, 2015 [14]	Male	60	Subacromial subdeltoid	Left	6 mo	Poly-L-lactic acid anchor	Debridement and evacuation of RBs	No

Literature documenting RBs in the shoulder including information on sex, age, location, laterality, symptom duration (sx dur.), underlying diagnosis (underlying dx), treatment (tx), and recurrence (recur).

## Data Availability

The data that support the findings of this study are available from the corresponding author upon request.

## References

[1] Guo J. J., Wu K., Xu Y., Yang H. (2020). Hundreds of rice bodies in the subacromial-subdeltoid bursa: report of two cases and literature review. *BMC Musculoskeletal Disorders*.

[2] Joshi P. S. (2018). Severe sub-acromial bursitis with rice bodies in a patient with rheumatoid arthritis: a case report and review of literature. *Malaysian Orthopaedic Journal*.

[3] Popert A. J., Scott D. L., Wainwright A. C., Walton K. W., Williamson N., Chapman J. H. (1982). Frequency of occurrence, mode of development, and significance or rice bodies in rheumatoid joints. *Annals of the Rheumatic Diseases*.

[4] Chen A., Wong L.-Y., Sheu C.-Y., Chen B.-F. (2002). Distinguishing multiple rice body formation in chronic subacromial-subdeltoid bursitis from synovial chondromatosis. *Skeletal Radiology*.

[5] Gillijns M., Vandesande W. (2022). Rice bodies in the wrist. *Modern Rheumatology Case Reports*.

[6] Skelly D. L., Konieczko E. M., Ulrich J. (2023). Rice bodies in a shoulder bursa: a cadaveric and histologic case report. *Journal of Manual & Manipulative Therapy*.

[7] Steinfeld R., Rock M. G., Younge D. A., Cofield R. H. (1994). Massive subacromial bursitis with rice bodies. Report of three cases, one of which was bilateral. *Clinical Orthopaedics and Related Research*.

[8] Law T. C., Chong S. F., Lu P. P., Mak K. H. (1998). Bilateral subacromial bursitis with macroscopic rice bodies: ultrasound, CT and MR appearance. *Australasian Radiology*.

[9] Iyengar K., Manickavasagar T., Nadkarni J., Mansour P., Loh W. (2011). Bilateral recurrent wrist flexor tenosynovitis and rice body formation in a patient with sero-negative rheumatoid arthritis: a case report and review of literature. *International Journal of Surgery Case Reports*.

[10] Zmerly H., Moscato M., Akkawi I. (2022). Assessment and management of loose bodies in the knee joint and related disease: a narrative review. *Current Rheumatology Reviews*.

[11] Best C., Basu A., Sengupta M. (2015). Subacromial‐subdeltoid bursal rice bodies causing shoulder pain. *PM & R: the Journal of Injury, Function, and Rehabilitation*.

[12] Vyas S., Bhadu D., Goswami R. P., Kumar U. (2022). Subacromial subdeltoid rice body bursitis in rheumatoid arthritis treated with local steroids. *International Journal of Rheumatic Diseases*.

[13] Barad S. J. (2019). Severe subacromial-subdeltoid inflammation with rice bodies associated with implantation of a bio-inductive collagen scaffold after rotator cuff repair. *Journal of Shoulder and Elbow Surgery*.

[14] Sivaloganathan S., Amr R., Shrivastava R., Relwani J. (2015). The Risotto sign – a severe inflammatory bursitis with rice body formation, complicating a rotator cuff repair with a bioabsorbable suture anchor. *JRSM Open*.

[15] Cuomo A., Pirpiris M., Otsuka N. Y. (2006). Case report: biceps tenosynovial rice bodies. *Journal of Pediatric Orthopaedics B*.

[16] Tan C. H. A., Rai S. B., Chandy J. (2004). MRI appearances of multiple rice body formation in chronic subacromial and subdeltoid bursitis, in association with synovial chondromatosis. *Clinical Radiology*.

